# Emerging Roles of Inflammasomes in Cardiovascular Diseases

**DOI:** 10.3389/fimmu.2022.834289

**Published:** 2022-04-07

**Authors:** Yingnan Liao, Kui Liu, Liyuan Zhu

**Affiliations:** ^1^Xiamen Key Laboratory of Cardiovascular Disease, Xiamen Cardiovascular Hospital of Xiamen University, School of Medicine, Xiamen University, Xiamen, China; ^2^Institute of Pharmaceutical Science, China Pharmaceutical University, Nanjing, China

**Keywords:** inflammasome, atherosclerosis, myocardial infarction, heart failure, cardiac hypertrophy

## Abstract

Cardiovascular diseases are known as the leading cause of morbidity and mortality worldwide. As an innate immune signaling complex, inflammasomes can be activated by various cardiovascular risk factors and regulate the activation of caspase-1 and the production and secretion of proinflammatory cytokines such as IL-1β and IL-18. Accumulating evidence supports that inflammasomes play a pivotal role in the progression of atherosclerosis, myocardial infarction, and heart failure. The best-known inflammasomes are NLRP1, NLRP3, NLRC4, and AIM2 inflammasomes, among which NLRP3 inflammasome is the most widely studied in the immune response and disease development. This review focuses on the activation and regulation mechanism of inflammasomes, the role of inflammasomes in cardiovascular diseases, and the research progress of targeting NLRP3 inflammasome and IL-1β for related disease intervention.

## Highlights

1. Many cardiovascular diseases risk factors, such as obesity and cholesterol crystals (ox-LDL/ox-PLs), can trigger the activation of the inflammasome, thus inducing inflammation.
2. Active inflammasome, especially NLRP3 inflammasome, plays a pivotal role in the development of cardiovascular diseases, such as atherosclerosis, myocardial infarction, and heart failure.
3. As promising targets for cardiovascular diseases, NLRP3 and IL-1 inhibitors have achieved positive clinical effects; it is of great clinical significance to develop more novel and specific inhibitors.

## 1 Introduction

Although major improvements in treatment and outcomes, cardiovascular diseases (CVDs) remain the leading cause of morbidity and mortality worldwide (1). The primary clinical endpoints of CVDs are heart failure (HF) and CVD death, mainly due to myocardial infarction (MI), which is mainly attributable to atherothrombotic plaques occlusion of blood vessels. Long-term atherosclerotic changes are driven by dyslipidemia and vascular inflammation. Some environmental and genetic factors, including hypertension, diabetes, hyperlipidemia, smoking, and aging, have been linked with CVDs (2). However, the pathological process of CVDs, especially atherosclerosis (AS) and myocardial ischemia/reperfusion (MI/R) injury, is gradually considered to be closely related to the inflammatory response. Therefore, inflammatory pathways may be effective targets in the treatment of CVDs. In fact, several clinical studies have assessed the outcomes of anti-inflammatory therapy in patients with AS (3).

As a multi-protein complex assembled by intracytoplasmic pattern recognition receptors, inflammasome is an important part of the natural immune system. It has been determined that a variety of inflammasomes participate in the host defense response against various pathogens (4). It can recognize pathogen-related molecular patterns or host-derived damage-associated molecular patterns (DAMPs) and recruit and activate the caspase-1, a proinflammatory protease. Activated caspase-1 cleaves the cytokine precursors pro-interleukin-1beta (IL-1β) and pro-IL-18 to produce corresponding mature cytokines in the process of innate immune defense (5). The activation of inflammasome can also induce the caspase-1-dependent programmed cell apoptosis (pyroptosis) and cell death stimulated by inflammation and stress (6). Inflammasome activation results in a very powerful self-amplifying and inflammatory response and thus modulates a variety of inflammatory diseases, such as CVDs. A balanced inflammatory response promotes damage resolution and tissue healing, while excessive inflammasome activation leads to harmful effects such as tissue damage (7). The activation of the inflammasome is considered to be involved in the pathogenesis of various CVDs. The intervention of inflammasome-mediated signaling can reduce inflammation and the severity of diseases (8). Here we review the role and mechanism of NOD, LRR, and PYD domain-containing protein (NLRP) 1, NLRP3, NOD, LRR, and CARD domain-containing protein (NLRC) 4 and absent in melanoma 2 (AIM2) inflammasomes in regulating CVDs, including AS, myocardial ischemic injury, cardiac hypertrophy, and HF; we summarize the therapeutic effect of pharmacological intervention targeting NLRP3 inflammasome and IL-1β in the experimental and clinical studies. In particular, as the core of inflammatory response, a crucial involvement of the NLRP3 inflammasome in CVDs has attracted widespread attention in the academic community in recent years and may provide a new therapeutic possibility for various inflammatory diseases (7). At present, the NLRP3 inflammasome is expected to become a new target for the prevention and treatment of CVDs (9). Issues for the activation and assembly of the NLRP3 inflammasome and the in-depth mechanism of mediating cell pyroptosis, the specific regulatory mechanism of the active ingredients for the NLRP3 inflammasome, and the changing characteristics of NLRP3 inflammasome under CVDs still need to be further studied.

## 2 Structure and Activation of Inflammasomes

### 2.1 Structure of Inflammasomes

The basic structure of most inflammasomes is based on the NOD-like receptor or ALR protein family as the receptor protein, apoptosis-associated speck-like protein containing CARD (ASC) as the adaptor protein, and pro-caspase as the effector protein (5).

The NLRP3 inflammasome consists of three components: NLRP3, ASC, and caspase-1. The innate immune receptor NLRP3 protein contains three domains: NACHT, a central nucleotide domain; leucine-rich repeats (LRR); and pyrin domain (PYD). Besides, the adaptor protein ASC contains two interaction domains: PYD and caspase recruitment domain (CARD). In the case of inflammasome assembly, the PYD domain interacts with the PYD domain of ASC, while the CARD structure of ASC can be combined with the CARD domain of the effector protein, during which the ASC acts as a bridge, connecting the receptor protein and the effector protein (4). Through this interaction, once the receptor protein is stimulated, caspase-1 can be finally activated and cleaved. Activated caspase-1 can cut and promote the maturation and release of IL-1β and IL-18 (10), thus causing inflammation. In addition, inflammasome activation can mediate caspase-1-dependent pyroptosis (6) and thus promote the spread of the inflammatory response.

AIM2 contains an N-terminal PYD domain, C-terminal HIN200 domain. AIM2 is a type of DNA sensor that can recognize and bind autologous or foreign DNA through its C-terminal HIN200 domain, which can be completed without relying on sequence (11). DNA sensing by AIM2 results in the assembly of inflammasome, which is a supramolecular multi-protein complex. The HIN200 domain of AIM2 binds to DNA, while the PYD domain (but not that of the other PYHIN family members) establishes interaction with the PYD domain of the adapter molecule ASC, and the CARD domain of ASC associates with the CARD domain of pro-caspase-1 to activate both NF-κB and caspase-1 (11), leading to the assembly of activated AIM2 inflammasome (12).

**Figure 1:**
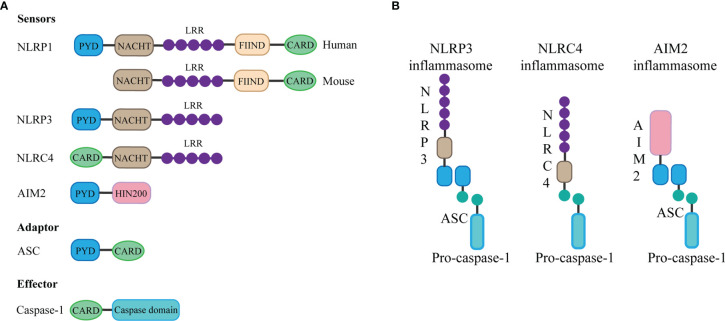
Components and structure of inflammasomes. **(A)** The basic structure of most inflammasomes is based on the NOD-like receptor or ALR protein family as the sensor protein, ASC as the adaptor protein, and pro-caspase as the effector protein. Different sensor proteins consist of different domains: PYD, NACHT, LRR, function-to-find domains (FIIND), CARD, and HIN domain. **(B)** The PYD domain of NLRP3 or AIM2 interacts with the PYD domain of ASC, while the CARD domain of ASC can be combined with the CARD domain of the effector protein pro-caspase-1 to form inflammasome. NLRC4 does not contain the PYD domain but contains the CARD domain, which means that it can directly connect through the CARD domain with the CARD domain of the effector protein.

The special feature of the NLRC4 inflammasome is it does not contain the PYD domain but contains the CARD domain, which means that it does not need the ASC protein as a “bridge” and can directly connect through the CARD domain with the CARD domain of the effector protein (13). The assembly of NLRC4 is accomplished through interaction with NOD-like receptor family apoptosis inhibitory proteins, and the invasion of typhoid fever is an agonist of this process (14). Besides, in contrast to human NLRP1, mouse NLRP1b protein also does not contain the PYD domain but contains the CARD domain, which can directly bind to the effector protein without ASC (15). The structure of the inflammasome is summarized in **Figure 1**.

### 2.2 Expression and Activation of Inflammasomes

Important components of inflammasome such as NLRP3 are not constructively expressed in cardiomyocytes but regulated by NF-κB (16, 17). Proinflammatory stimuli, such as cell debris or microbial products (referred to as DAMPs or pathogen-associated molecular patterns) (16), induce the expressions of NLRP3 and other inflammasome components in cardiomyocytes, leukocytes, fibroblasts, and endothelial cells (ECs) (18, 19). In the initial stages of formation, the inflammasome specks mainly accumulate in the ECs, cardiomyocytes, and fibroblasts. As the leukocytes infiltrate the heart, the majority of specks can be seen within the granulocytes and macrophages. In the post-injury healing phase, the majority of specks are more common in isolated cardiomyocytes or fibroblasts (20, 21).

Although each type of cell in the heart may trigger the NLRP3 inflammasome with appropriate stimulation, the stimulation required and the role of the active inflammasome may differ. Circulating monocytes constitutively express many components of inflammasome and produce large amounts of IL-1β with minimal stimulation (22). Conversely, cardiomyocytes express very low and almost undetectable levels of IL-1β, which is unaffected even in response to high doses of proinflammatory mediators. Besides, cardiomyocytes have low expressions of NLRP3 and caspase-1. However, both monocytes and cardiomyocytes can express and activate IL-18, and cardiomyocytes die of pyroptosis after caspase-1 activation (16, 22). The difference between the secretion of IL-1β and IL-18 secretion may be related to their transcriptional regulation (23).

Activation of NLRP3 inflammasome requires two separate steps: priming and triggering (17). A trigger is required after the activation is completed and the NLRP3 inflammasome components are expressed, which is largely dependent on intracellular K^+^ concentration. Lysosome instability activates a signaling pathway that indirectly leads to increased membrane permeability for K^+^ and intracellular efflux (16). Extracellular ATP triggers K^+^ outflow binding to the purinoreceptor 7 (P2X7). A change in K^+^ concentration results in a conformational change in NLRP3, thus leading to the recruitment of ASC (16). The use of transgenic mice with constructive NLRP3 activity showed that NLRP3 activation alone was not sufficient to cause cardiac dysfunction (17). Furthermore, patients with cryopyrin-associated periodic symptoms, who have mutations in NLRP3 gene causing continued NLRP3 activity, usually do not appear to have cardiac dysfunction but may be more susceptible to cardiac dysfunction during clinically active phases, proving the indispensability of priming for the activation of NLRP3 inflammasome (24).

The time-dependent activation of the NLRP3 inflammasome in the heart has been studied using animal models. In a model of ischemia–reperfusion injury, the expression of NLRP3 and the activity of the inflammasome in the heart were low within 3 h after acute MI (AMI), significantly increased in the myocardium within 3–24 h, and peaked after 1 and 3 days in mice with reperfused and non-reperfused AMI, respectively, aggravating inflammatory response and the ischemia–reperfusion injury (16, 18, 20, 21, 25). Therefore, delayed treatment with NLRP3 inhibitor (16673-34-0) even by 60 min after reperfusion initiation still exerted its cardioprotective effect, while the protective effect was lost if administered more than 3 h after reperfusion (20).

Active NLRP3 inflammasome enhances the recruitment and activation of caspase-1. The activated caspase-1 promotes the activation of the precursors of IL-1β and IL-18 to IL-1β and IL-18 as well as the cleavage of gasdermin D (GSDMD) (26). The cleaved GSDMD then forms pores on the cell membrane, triggering proinflammatory cell death, that is, pyroptosis (27). This process is accompanied by the release of IL-1β, IL-18, and alarm elements such as high mobility group proteins 1, which spreads dangerous signals from damaged or dead cells and mobilizes immune cells, especially in the recruitment of neutrophils (28). In addition, oligomeric inflammasome particles can be phagocytosed by surrounding macrophages to amplify the inflammatory response (29).

**Figure 2:**
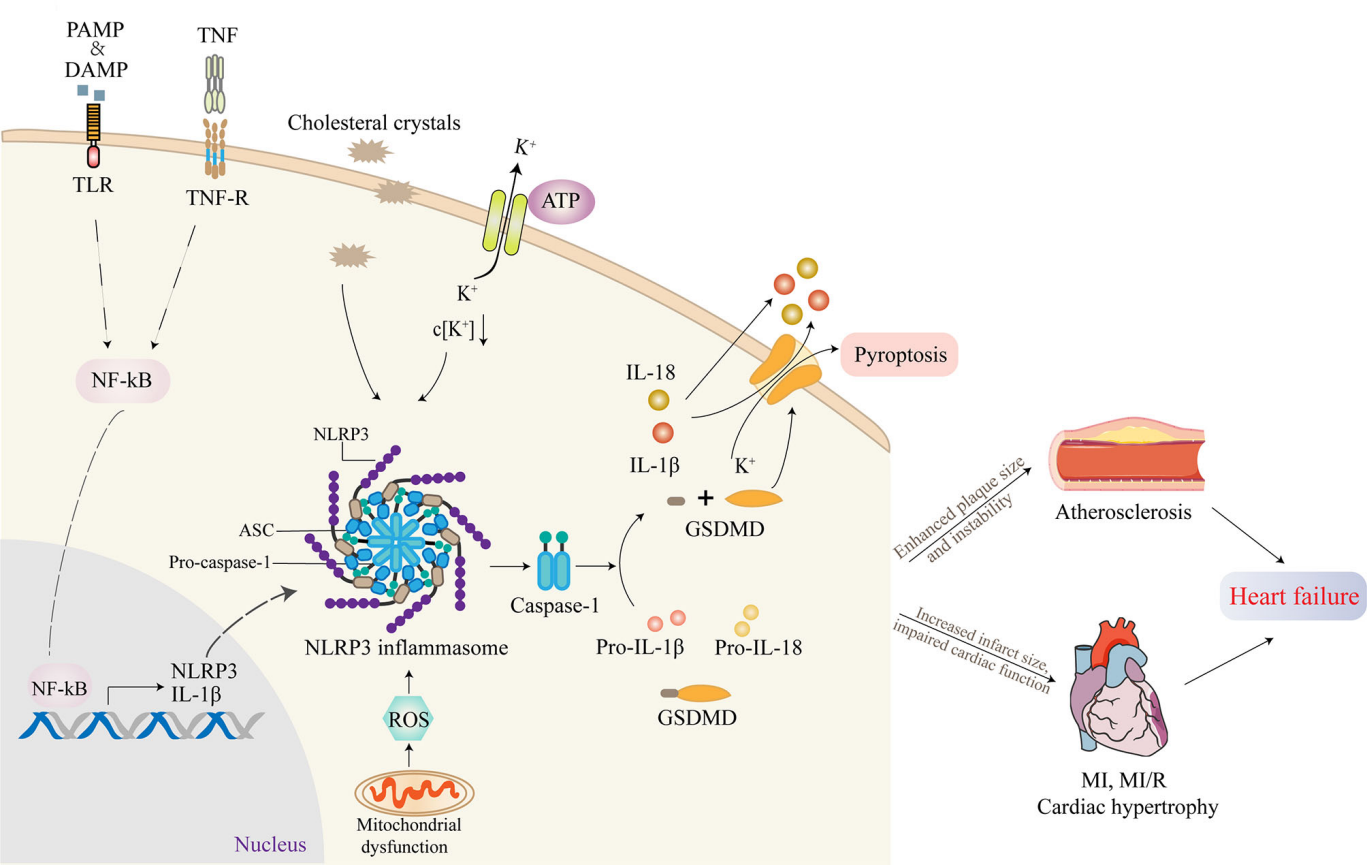
The activation of NLRP3 inflammasome and its role in cardiovascular diseases (CVDs). Inflammasome activation requires two independent steps: priming and triggering. Proinflammatory stimuli, such as PAMP, DAMP, or TNF, induce NF-κB signaling through TLR, TNF-R. The transcription factor NF-κB translocates to the nucleus and induces the expression of the IL-1β precursor pro-IL-1β and NLRP3. Cholesterol crystals or ox-LDL can be phagocytosed through receptor-mediated phagocytosis, leading to the formation of cholesterol crystals in phagolysosomes. Another mechanism of NLRP3 activation involves crystal-induced lysosomal rupture, or ATP-gated P2X7R-mediated decreased intracellular K^+^ concentration can activate NLRP3 protein. Once activated, NLRP3 recruits adapter molecule ASC, which interacts with pro-caspase-1, resulting in the formation of NLRP3 inflammasome. Active NLRP3 inflammasome promotes the recruitment and activation of caspase-1, which is responsible for the activation of IL-1β and IL-18 as well as the cleavage of GSDMD. The cleaved GSDMD then forms pores on the cell membrane, triggering pyroptosis. Most studies supported that the activation of NLRP3 inflammasome contributed to the development of atherosclerosis (AS), myocardial infarction (MI), myocardial ischemia/reperfusion (MI/R), and cardiac hypertrophy, which ultimately promoted heart failure.

NLRP3 inflammasome-activating ligands can stimulate the production of reactive oxygen species (ROS), which in turn promote the assembly of inflammasome (30). Consistently, Jurg Tschopp found that mitochondrial-derived ROS are the key signal that regulates the activation of NLRP3 inflammasome (31). At the same time, it was found that autophagy and mitophagy regulate the quality of mitochondria and reduce the number of damaged mitochondria, thus preventing the activation of NLRP3 inflammasome induced by ROS. Autophagy-deficient cells exacerbate the activation of NLRP3 inflammasome. Further, the voltage-dependent anion channels of mitochondria regulate the production of mitochondrial ROS and the activation of NLRP3 inflammasome (31). The mechanism of how ROS triggers the activation of NLRP3 remains to be further investigated (30). The activation process of NLRP3 inflammasome is summarized in **Figure 2**.

Research has shown that a variety of risk factors for CVDs, obesity, diabetes, and metabolic syndrome can activate NLRP3 inflammasome and promote inflammation (32, 33). Compared with healthy adults, the expressions of the NLRP3 inflammasome, ASC, caspase-1, IL-1β, and IL-18 in the serum of patients with coronary heart disease were significantly increased (34). Elevated levels of *NLRP3* mRNA in cardiac hypertrophy, inflammatory response, and ventricular dilatation were observed in mouse models (35). The expression and release of mediators such as IL-1β and IL-18 promote the occurrence and development of AS and affect the stability of AS (36, 37), suggesting their important role in the pathogenesis of CVDs. Intervention in the formation and activation of NLRP3 inflammasome is expected to provide new ideas for the prevention and treatment of CVDs. Inhibition of the NLRP3/IL-1β pathway can alleviate cardiac inflammatory response, improve cardiac contractility, and attenuate cardiomyopathy in sepsis (38). Therefore, it is speculated that the immune pathway mediated by inflammasome is involved in the development of ventricular remodeling in patients with coronary heart disease (39).

So far, there have been conflicting results in research on the potential association between the polymorphism of *NLRP3* and CVDs. A study showed that *NLRP3 rs10754558* polymorphism is associated with the occurrence and severity of coronary artery disease and the increase in serum concentration of IL-1β (40). On the contrary, another study revealed no association between *NLRP3* polymorphism and CVDs (41). However, the cohort sizes of the two studies are relatively small, so larger studies involving more patients will be needed in the future to accurately evaluate the impact of *NLRP3* polymorphisms on CVDs.

## 3 Inflammasomes in Cardiovascular Diseases

### 3.1 Activation of Inflammasomes in Cardiovascular Diseases

AS is a progressive disease of the arteries, characterized by inflammation of the blood vessel walls and the accumulation of cholesterol, calcium, and cellular debris. Cholesterol crystals (ChCs), a major component of atherosclerotic lesions, are known to directly activate NLRP3 inflammasome by inducing the infiltration of lysosomal protease cathepsin B into the cytoplasm (42), thus inducing a series of inflammation *in vivo* and *in vitro*, which links lipid metabolism and inflammation. ChCs were also found to trigger NLRP3 activation in human macrophages and mouse carotid arterial ECs (43). Moreover, ChCs induced severe endothelial dysfunction in mouse coronary arteries, which can be alleviated in *Nlrp3−/−* mice (44). Besides, it has been found that oxidized low-density lipoprotein (ox-LDL) upregulated the protein levels of NLRP3, caspase-1, and IL-1β in vascular ECs and increased cell pyroptosis, *via* inhibiting tetmethylcytosine dioxygenase 2, which can be downregulated by blocking the NF-κB pathway and improving mitochondrial functions (45). Oxidized phospholipids (oxPLs), a product of lipid peroxidation, mainly result from tissue injury and lead to proinflammatory or anti-inflammatory responses, depending on the context (46), which have been reported to potentiate oxidative stress, trigger sterile inflammation, and be involved in many infectious and sterile-mediated inflammation in the CVDs, including AS (47). The levels of oxPLs are increased in hyperlipidemic human plasma and atherosclerotic lesions (48). Ox-LDL and oxPLs increase oxidative stress in the endothelium and promote the activation of NLRP3 inflammasome and release of IL-1β, thereby contributing to the atherogenic phenotype of ECs (49). Significantly increased oxPLs were observed *in vivo* and *in vitro* following MI/R. Furthermore, oxPLs resulted in rat cardiomyocyte cell death *in vitro*, and neutralization of oxPL *in vivo* reduced infarct size after MI/R, which may represent a novel potential therapeutic avenue to attenuate I/R injury and improve cardiac outcomes during AMI (50). Therefore, based on the association between the activation of inflammasome, especially NLRP3, and atherogenic factors, whether the inflammasome might be involved in AS has been extensively studied (51, 52).

Actually, several epidemiological studies have linked aortic NLRP3 expression with CVD prevalence, thus indirectly confirming the importance of the NLRP3 inflammasome in humans. The levels of NLRP3 in aortic (53) and peripheral blood monocytes (54) were elevated in patients with coronary AS and positively correlated with the disease severity and the atherosclerotic risk factors, such as hypertension, smoking, LDL-C, and high-density lipoprotein-C levels, which are possible to be predictors of major adverse cardiac events. Furthermore, NLRP3, ASC, caspase-1, IL-1β, and IL-18 were highly expressed in human carotid atherosclerotic plaques, compared with healthy mesenteric arteries or iliac arteries (55, 56), and the expression levels were even higher in unstable in comparison to stable plaques (55).

The cell debris released during AMI functions as DAMPs, which induces the expressions of key components of the inflammasome through activation of the NF-κB or cell surface receptors, such as IL-1R1 (17). Compared with healthy controls, the NLRP3 and caspase-1 mRNA transcription and protein levels of circulating monocytes in patients with acute coronary syndrome or stable angina pectoris are increased (23).

The expressions of NLRP1 and NLRC4 were increased in patients with primary AS lesions compared with healthy controls, and inflammasome complex was activated by interaction with NLRP1 and NLRC4 receptors (57). Another study has reported that elevated levels of plasma triglycerides and very-low-density lipoprotein cholesterol of patients with AS, manifested as peripheral arterial disease, promoted activation of the NLRP1 inflammasome by NF-κB in human arterial ECs (58). It is suggested that statin can regulate the expression of NLRP1 inflammasome *via* sterol regulatory-element binding proteins (59). It has been reported that the NLRP1 inflammation pathway is activated after MI, which can be suppressed by the promotion of autophagic flux (60). More and more evidence suggests that NLRP1 may trigger the immune-inflammatory processes in arterial ECs and affect the vessel remodeling; thus, NLRP1 inflammasome inhibition may be a novel therapeutic approach to peripheral arterial disease (61). A genome-wide association study showed that the NLRC4 inflammasome was important for IL-18 production in acute coronary syndrome patients, thus promoting the formation of atherosclerotic plaque (62).

Notably, AIM2 was constitutively expressed in normal intima and media of the human carotid artery as well as the aorta and was upregulated around the necrotic core of human atherosclerotic lesions (63). Abundant AIM2 expression was associated with increased dsDNA deposition in late AS, during which dsDNA released by necrotic cells may activate the AIM2 inflammasome and promote the release of atherosclerotic cytokines, and in return, AIM2-mediated cell death may contribute to the deposition of dsDNA in the necrotic core (64). AIM2 and NLRC4 inflammasomes in cardiac macrophages and cardiomyocytes were found to be increased in the area around left ventricle (LV) infarction, thus resulting in cardiomyocyte death and HF and confirming the previous finding that NLRC4 plays an important role in regulating IL-18 in patients with acute coronary syndromes (62, 65) It has been found that expressions of the inflammasome protein AIM2 and NLRC4 were increased in human HF samples, while the NLRP1/NALP1 and NLRP3 inflammasome showed no change, among which the expression of AIM2 was primarily detected in monocytes/macrophages of failing hearts and has been further verified in animal models (66).

As the important downstream effector of the inflammasome, IL-1 and IL-18 play an important role in AS. IL-1 was shown to be expressed in human atherosclerotic plaques (67) and contribute to the initiation, formation, growth, and rupture of the plaques (68, 69). Animal studies have shown that IL-1β expression levels were elevated in animals with ventricular remodeling (70). The levels of IL-18 were upregulated in mice with either MI/R injury or MI (71, 72). A study reported that plasma levels of IL-18 were increased in patients with congestive HF, which was directly associated with the severity of myocardial damage and dysfunction (73).

In short, many CVD risk factors can lead to the activation of the inflammasome and its downstream molecules, which in turn participate in the pathological process of the disease.

### 3.2 Experimental Evidence for the Role of Inflammasomes in Cardiovascular Diseases

#### 3.2.1 Atherosclerosis

The role and mechanism of inflammasomes in AS have been studied extensively through animal experiments. Latz and colleagues found that in mice lacking *Ldlr*, inflammasomes contributed to the progression of AS, and mice lacking *Nlrp3* or *Il-1* were resistant to diet-induced systemic inflammatory cytokine responses and AS (51, 74). Consistently, transplantation with *Nlrp3^−/−^*, *Asc^−/−^*, or *Il-1α/β^−/−^* bone marrow into *Ldlr^−/−^* mice markedly decreased early AS in parallel with the reduction in IL-1β and IL-18 levels (51). Moreover, knockdown of *Nlrp3* by lentiviral silencing (75) in *ApoE^−/−^* mice prevented AS progression and reduced the production of proinflammatory cytokines and the levels of plasma lipids with an increase of macrophage polarization from the M1 to M2 state, which further confirms the importance of NLRP3 inflammasome. It has been found that the NLRP3 inflammasome not only can mediate the early development of early AS but also can promote the formation of vulnerable plaques (76). Thus, the response of inflammasome to ChCs may be a key early trigger of atherosclerotic inflammation.

Cholesterol efflux is mediated by the cholesterol transporters ATP-binding cassette A1 and G1 (ABCA1/G1), transforming cholesterol into high-density lipoprotein, which plays a fundamental role in atherogenesis (77). Bone marrow transplantation from mice with myeloid *Abca1/g1^−/−^* along with *Nlrp3* or *caspase-1/11* double knockout into *Ldlr^−/−^* mice fed with a western diet suggested that *Nlrp3* and *caspase-1/11* deficiency inhibited atherosclerotic lesions in myeloid *Abca1/g1*-deficient *Ldlr^−/−^* mice. The study also showed that inflammasome activation enhanced neutrophil recruitment and neutrophil extracellular trap formation in plaques (78).

Regulating the caspase-1 can help reverse the progression of AS and maintain plaque stability. Two independent studies revealed that deficiency of caspase-1 in *ApoE^−/−^* mice showed reduced atherosclerotic lesions fed with a lower cholesterol atherogenic diet (79) or a decreased spontaneous development of lesions with a chow diet for 26 weeks (80). Another study (81) has found that bone marrow transplantation of *caspase-1/11^−/−^* mice into *Ldlr^−/−^* mice showed a strong reduction in atherosclerotic plaque size, as well as necrotic core content compared with control bone marrow. In a word, these studies indicate that the accumulation of lipids (mainly ox-LDL and ChCs) induces the activation of the NLRP3 inflammasome, which then promotes monocyte adhesion, macrophage transformation into foam cells, and the subsequent development of atherosclerotic lesions (82).

Moreover, in atherosclerotic plaques of *ApoE^−/−^* mice, purinergic receptor P2X7 is co-expressed with NLRP3 and plays an important role in AS by promoting phosphorylation of protein kinase R to activate NLRP3 inflammasome (83). Interestingly, microbial pathogens promote AS *via the* NLRP3 inflammasome. For example, *Nlrp3* deficiency inhibited the *Chlamydia pneumoniae*-accelerated AS (84). Similarly, *Porphyromonas gingivalis*, a periodontal disease pathogen, activated NLRP3 inflammasome *via* interacting with pattern recognition receptors and augmented atherogenesis (85).

The activation of the NLRP3 inflammasome is affected by multiple signaling pathways. Therefore, differences in the systemic or local environment may influence the gene dose effect. It is suggested that deletion of *Nlrp3* in *Ldlr^−/−^* mice inhibited the development of AS due to other pathways participating in the local inflammation, while *Nlrp3* deletion alone only had a small effect (78). Besides, another study showed that *Nlrp3*, *Asc*, or *caspase-1* deletion did not show a reduction in AS development in ApoE-deficient mice fed a high-fat diet (86), while caspase-1 and *Cd36* deletion significantly inhibited AS (79, 80, 87), suggesting that the development of atherogenesis may not depend on NLRP3 inflammasome activation (88). These conflicts may be explained by the differences in experimental conditions, including the mouse model, gender, age, the type of atherogenic diet, and high-fat diet feeding time. Actually, *ApoE^−/−^* mice used in this study (86) develop diet-induced AS faster and more severely than *Ldlr^−/−^* mice used in Latz’s study (51). Moreover, the mice were fed with a stronger atherogenic diet with more than 8-fold higher cholesterol content for 11 weeks, which was 3 weeks longer than in the other study (51). It is possible that excessive dietary cholesterol combined with a prolonged feeding period triggers other inflammatory pathways, potentially weakening the role of NLRP3 inflammasome in AS development. More in-depth studies are needed in the future to clarify the role of the NLRP3 inflammasome.

A recent study (64) has explored the function of the AIM2 inflammasome in AS using *ApoE^−/−^* mice and found that *Aim2* deletion significantly reduced IL-1β and IL-18 levels within plaques, enhanced atherosclerotic plaque stability, and improved the histopathological features after a high-fat diet. Clonal hematopoiesis, which is very common among the elderly, increases the risk of MI and stroke independent of traditional risk factors, among which the *Jak2^V617F^* (*Jak2^VF^*) mutations pose the greatest risk of premature coronary heart disease (89, 90). Jak2^VF^ lesions lead to DNA replication stress and activation of the AIM2 inflammasome, thereby aggravating AS, while *Aim2* deficiency inhibited AS (91). The mechanism of AIM2 regulating AS involves the following aspects. First, overexpression of *Aim2* promoted plaque lesion formation and increased intracellular adhesion molecule-1 (ICAM-1) expression (92), which contributed to monocyte recruitment into arterial endothelium at AS-prone areas through binding lymphocyte function-associated antigen-1. Deletion of *Icam-1* in mice reduced aortic lesion size (93). Therefore, increased ICAM-1 level may represent a mechanism for AIM2-induced AS. Second, with the stimulus of ox-LDL, AIM2 had the ability to trigger vascular smooth muscle cell (VSMC) migration and pyroptosis, which are known to be crucial drivers of early AS (94). An *in vitro* study showed that SMC recruitment and migration were enhanced by *Aim2* overexpression and blunted by *Aim2* silencing, possibly through regulation of TGF-β/Smad2 signaling and matrix metalloproteinase-2, a key regulator of VSMC migration, providing a mechanism for AIM2 involvement in AS (95). Besides, it has been found that AIM2 mediated VSMC pyroptosis and GSDMD activity through ASC, caspase-1 pathway (92). Third, AIM2 increased the release of proinflammatory cytokines such as IL-1β and IL-18 (64), which were important contributors to the development of lesions, thereby promoting inflammation in atherosclerotic lesions.

Aside from the direct role in AS, AIM2 also has an indirect effect on atherosclerosis. AIM2 induces non-canonical activation of NLRP3 inflammasome *via* AIM2 inflammasome-mediated pore formation in the cell membrane and subsequent K^+^ efflux (96). Given the known role of NLRP3 inflammasome in atherosclerosis, it is possible that AIM2 promotes the development of atherosclerosis partially through activating the NLRP3 inflammasome (97).

As downstream effectors of inflammasomes, it has been reported that IL-1α played a key role in the early stage of atherosclerosis, while IL-1β induced inflammation in advanced atherosclerosis in mice (98). Il-1 receptor antagonist (*Il-1ra*) deficient in *ApoE* knockout mice developed atheromatous plaques treated with a high-fat diet (68). Consistently, deletion of *Il-1r1* in *ApoE*-deficient mice showed a reduction of atherosclerotic plaques in the aortic root, but not in the brachiocephalic artery (99). *Il-1β* deficiency reduced atherosclerotic lesions in *ApoE^−/−^* mice (100) and *Ldlr^−/−^* mice transplanted with bone marrow of *Il-1α/Il-1β*-deficient mice showed impaired diet-induced atherosclerosis (51). Moreover, IL-18 is expressed in human atheroma cells, such as vascular ECs, SMCs, and macrophages, and also participated in the development of atherosclerosis (101). *Il-18* deficiency reduced the development of atherosclerosis in *ApoE^−/−^* mice (102). In brief, activated inflammasomes contribute to the development of AS; targeting inflammasomes may be a promising treatment for AS.

#### 3.2.2 Myocardial Infarction

Inflammasome formation is an energy-consuming process that occurs in injured cells and rapidly induces inflammation, which resembles the defense process. However, in the context of sterile inflammation, this inflammation may lead to further injury (16). The evidence that the NLRP3 inflammasome plays an essential role in the development of MI, adverse remodeling, and HF includes the upregulated inflammasome components, the reduced damage caused by inflammasome inhibition, and the involvement of effector molecules (IL-1β and IL-18) in the pathogenesis of MI and HF (8). Endogenous IL-1β and IL-18 also mediated contractile dysfunction following myocardial ischemia (103). Many experimental studies have explored whether the formation of the inflammasome in the injured cardiomyocytes is beneficial or harmful with further injury.

Studies have shown that I/R enhanced NLRP3 inflammasome activation in cardiac microvascular ECs but not cardiomyocytes, and deficiency of *Txnip*, a contributor to I/R injury, reduced NLRP3 inflammasome activation and infarct size in mouse myocardial tissues (104). Besides, NLRP3 inflammasome is significantly increased in myocardial fibroblasts post-MI, which is essential for myocardial injury. ATP and K^+^ efflux have been shown to promote NLRP3 inflammasome assembly and IL-1β secretion in cardiac fibroblasts, while a marked reduction of infarct size and improvement of cardiac function were observed in hearts from *Nlrp3*-deficient mice after *ex vivo* I/R, but not in *Asc*-deficient hearts (105), suggesting that NLRP3 inflammasome may be an important contributor to hypoxic damage during ischemia–reperfusion. Consistently, a study has shown that NLRP3 inflammasome-mediated pyroptosis aggravates MI/R injury in diabetic rats (106). *Nlrp3* siRNA attenuated macrophage and neutrophil infiltration, cardiomyocyte apoptosis, and infarct size, thus inhibiting MI/R injury (105). Deletion of *Asc* or *caspase-1* reduced myocardial levels of active IL-1β and diminished infarct area and myocardial fibrosis and dysfunction after MI/R injury (19).

However, the role of NLRP3 in AMI is also time-dependent, and *Nlrp3* deficiency did not play a protective role during the first few hours of AMI due to low cardiac expression at the onset of ischemia, which was consistent with the 2-step process required for activation (20, 107). Besides, another study showed that deficiency of *Nlrp3* increased myocardial infarct size after MI/R injury *in vivo*, possibly due to dysfunction of the cardioprotective RISK pathway (21). The effect of NLRP3 inflammasome on myocardial injury warrants intensive study.

Taken together, most studies supported that NLRP3 inflammasome can mediate and amplify the inflammatory response and cell pyroptosis, thus aggravating myocardium damage and promoting ventricular remodeling after ischemia with or without reperfused myocardium. Regulating NLRP3 inflammasome can reduce MI/R injury and the area of ​​MI.

Activation of the AIM2 inflammasome is considered to be associated with increased numbers of cardiac M1 macrophages and infarct size, as well as with impaired LV function following MI (65). *MicroRNA(miR)-219a* attenuated cardiomyocyte apoptosis in a mouse model of MI/R *via* blocking the AIM2/TLR4 pathway, while overexpression of *Aim2* aggravated MI/R injury alleviated by *miR-219a (* 108). So it is assumed that AIM2 inflammasome contributes to MI/R injury probably by promoting the release of IL-18. Collectively, these findings suggest that activation of the AIM2 inflammasome and subsequent release of IL-18 may be a mechanism to exacerbate myocardial damage, LV dysfunction, and LV remodeling after MI.

Besides, NLRP1 inflammasome can be activated by ER stress *via* the NF-κB signaling pathway and promote MI/R injury. Knockdown of *Nlrp1* notably increased cell viability and suppressed hypoxia/reoxygenation-induced cell apoptosis, creatine kinase activity, and lactate dehydrogenase release (109), suggesting the important role of NLRP1 inflammasome in promoting myocardial ischemic injury. Therefore, inhibition of inflammasome activation may be new targets for the prevention and treatment of MI and subsequent HF.

#### 3.2.3 Cardiac Hypertrophy and Heart Failure

HF is the final stage of many cardiac dysfunctions and is the main cause of CVD death. Serum from patients with acute decompensated systolic HF suppressed contractility *via* an IL-1-dependent and IL-18-dependent mechanism (110). Regulation of NLRP3 inflammasome can decrease cardiac hypertrophy, improve cardiac diastolic and contraction function, and inhibit the development of HF. The study has shown that *Nlrp3* knockout or NLRP3 inflammasome inhibition by MCC reduced macrophage accumulation and cardiac fibrosis during AngII infusion, similar to the effect of Ca^2+^/calmodulin-dependent protein kinase II (*CaMKII*) *δ* deletion (111). Furthermore, it has been found that in a mouse model of transverse aortic constriction (TAC), expression of NLRP3 inflammasome was increased in the heart, and cardiomyocyte-specific *CaMKIIδ* deletion decreased NLRP3 inflammasome levels in cardiomyocytes, assessed by caspase-1 activity and IL-18 activation, inhibited ventricular dilation and contractile dysfunction, and reduced cardiac fibrosis and remodeling, indicating that CaMKIIδ stimulates NLRP3 inflammasome activation in response to pressure overload (112).

However, another study revealed that *Nlrp3* deletion impaired cardiac function and that enhanced inflammation, fibrosis, and hypertrophy increased Toll-like receptor 4 expressions in a mouse model of pressure overload interruption, suggesting a negative role of NLRP3 in regulating cardiac remodeling (113). Interestingly, it has been clarified that diabetes further worsened cardiac function and increased HF by promoting the activation of AIM2 and NLRC4 inflammasomes, but not NLRP3 inflammasome (65). Therefore, further investigation is needed to clarify the exact role of the NLRP3 inflammasome.

It has been studied that *Nlrp1* deficiency significantly inhibited aortic banding-induced cardiac hypertrophy, inflammation, and fibrosis *in vivo* and protected against isoproterenol-induced cardiomyocyte hypertrophy *in vitro*, which was associated with inhibited MAPK, NF-κB, and TGF-β/Smad pathways, indicative of the key role of NLRP1 in the development of HF (114).

Diabetic cardiomyopathy is an important cause of HF. An increased AIM2 expression was observed in the streptozotocin-induced diabetic rat model. Silencing of *Aim2* decreased diameter of cardiomyocytes and collagen I and collagen III protein levels, attenuated cardiac dysfunction *in vivo*, and reduced GSDMD-NT-related pyroptosis *in vitro*, suggesting that AIM2 promotes diabetic cardiomyopathy by inducing pyroptotic cardiac cell death and ventricular remodeling (115).

Furthermore, as the downstream effector of the inflammasome (including NLRP3 and AIM2), the role of IL-18 in HF has been widely investigated. The study has suggested that IL-18 can cause myocardial action potential prolongation, decrease of K^+^ current, and increase of calcium activation in diabetic mice and then promote spontaneous contraction of cardiomyocytes through the oxidation and phosphorylation of CaMKII (112). Another clinical study (116) has shown that IL-18 can ultimately lead to HF by promoting myocardial fibrosis and ventricular remodeling. Besides, IL-18 also participated in the cardiomyocyte dysfunction stimulated by IL-1β (110).

Taken together, these findings suggest that inflammasomes play a detrimental role in the progression of CVDs and provide new insights into the molecular mechanisms underlying vascular inflammation (**Table 1**). It will be an interesting follow-up question clarifying the precise regulatory mechanism of inflammasome activation and downstream genes in specific CVDs.

**Table 1:** Experimental evidence on the role of inflammasomes in animal models of CVDs.

Genotype	Cell type	CVD model	Outcomes	Ref.
*Nlrp3^−/−^*, *Asc^−/−^*, *Il-1α/β^−/−^*	Bone marrow transplanted into *Ldlr^−/−^* mice	HFD for 8 weeks	Reduced aortic lesion area and IL-18 levels	(51)
*Nlrp3^−/−^Ldlr^−/−^*	Global	WD feeding for 8 weeks	Reduced inflammatory responses and atherosclerotic plaque size	(74)
*Nlrp3^−/−^Abca1/g1^−/−^*, *Caspase-1/11^−/−^Anca1/g1^−/−^*	Bone marrow transplanted into *Ldlr^−/−^* mice	HFD for 8 or 12 weeks	Reduced neutrophil accumulation and extracellular trap formation, decreased AS lesion size	(78)
*Nlrp3^−/−^Apoe^−/−^*, *Asc^−/−^Apoe^−/−^*, *Caspase1^−/−^Apoe^−/−^*	Global	HFD for 11 weeks	No effect on AS progression, macrophage infiltration, plaque stability	(86)
*Apoe^−/−^*	Lentivirus-mediated *Nlrp3* shRNA	HFD for 8 weeks	Prevented plaque progression and inhibited proinflammatory cytokines	(75)
*Caspase-1^−/−^Apoe^−/−^*	Global	HFD for 12 weeks	Reduced inflammatory cytokines, macrophage, and smooth muscle cell accumulation and plaque area	(79)
*Caspase-1^−/−^Apoe^−/−^*	Global	HFD for 8 weeks or low-fat diet for 26 weeks	Reduced the inflammatory status of lesions and inhibited AS	(80)
*Caspase-1/11^−/−^*	Bone marrow transplanted into *Ldlr^−/−^* mice	HFD for 12 weeks	Increased anti-inflammatory leukocytes and reduced AS plaque size	(81)
*Aim2^−/−^Apoe^−/−^*	global	HFD for 16 weeks	Enhanced plaque stability and improved the histopathological features	(64)
*Apoe^−/−^*	Overexpression with *Aim2* lentivirus; or inhibition with *Aim2* shRNA	HFD for 12 or weeks	AIM2 overexpression increased, while knockdown reduced plaque lesion area and migration and pyroptosis of smooth muscle cell	(92, 95)
*Il-1ra^−/−^Apoe^+/−^*	Global	HFD for 16 weeks	Increased AS lesion size	(68)
*Il-1r1^−/−^Apoe^−/−^*	Global	HFD for 27-30 weeks	Reduced plaque size and outward remodeling, and enhanced plaque instability	(99)
*Il-1β^−/−^Apoe^−/−^*	Global	HFD for 12 or 24 weeks	Reduced AS lesions with reduced expressions of VCAM-1 and monocyte chemotactic protein-1	(100)
*Il-18^−/−^Apoe^−/−^*	Global	HFD for 16 weeks	Reduced atherosclerosis and Th1 activity	(102)
*Nlrp3^−/−^*, *Asc^−/−^*	Global	MI 30 min, R 24 h	*Nlrp3*^−/−^ increased, while *Asc*^−/−^ had no effect on infarct size	(21)
*Nlrp3^−/−^*, *Asc^−/−^*	Global	*Ex vivo* cardiac I/R	Improved cardiac function and reduced hypoxic damage	(105)
*Nlrp3^−/−^*	Global	MI 30 min, R 3 h	Had no effect on infarct size	(107)
*Asc^−/−^*, *Caspase-1^−/−^*	Global	MI/R	Reduced myocardial IL-1β, inflammation, and infarct size	(19)
C57 BL/6 mice	*Nlrp1* siRNA	Hypoxia 1 h, reoxygenation 4 h	Suppressed Hypoxia/Reoxygenation-induced cardiomyocyte injury *in vitro*	(109)
*Nlrp3^−/−^*	Global	Aortic binding	Accelerated cardiac hypertrophy, fibrosis, inflammation, and impaired cardiac function	(113)
*Nlrp1^−/−^*	Global	Aortic binding	Inhibited cardiac hypertrophy, inflammation, and fibrosis	(114)
Sprague–Dawley rats	*Aim2* shRNA	Diabetic rats	Prevented myocardial fibrosis and cardiac dysfunction	(115)
*Il-18^−/−^*, *Il-18r^−/−^*	Global	IL-1β treatment	Prevented IL-1β-induced LV systolic dysfunction	(110)

The species not specified in the table are all mice.

CVDs, cardiovascular diseases; HFD, high-fat diet; WD, western diet; AS, atherosclerosis; MI, myocardial infarction; MI/R, myocardial ischemia/reperfusion; LV, left ventricle.

### 3.3 Pharmacological Intervention of Inflammasomes for Cardiovascular Diseases

Based on the important role of inflammasomes, a variety of studies have explored pharmacological interventions to treat cardiovascular diseases. NLRP3 inhibitor significantly decreased inflammatory cytokines and inhibited the development of atherosclerosis in *ApoE^−/−^* mice (117, 118). Marchetti and colleagues designed a novel inhibitor 16673-34-0 derived from glyburide that selectively inhibits NLRP3. This NLRP3 inhibitor significantly reduced infarct size and protected cardiac function after ischemia or reperfusion when given as single or repeated doses, demonstrating the crucial role of NLRP3 inflammasome in MI and I/R models (119, 120). Moreover, MCC950, a known anti-inflammatory small molecule, inhibits the binding of NLRP3 to ASC in mice *in vivo*, independent of ATPase activity of NLRP3 (121). Treatment with MCC950 decreased infarct size and preserved cardiac function in a pig MI model (20, 122). Consistently, it has been suggested that hematopoietic or myeloid *ten-eleven translocation 2* deficiency elevated IL-1β expression and enhanced TAC-induced cardiac remodeling and cardiac dysfunction, thus promoting the development of HF, which was rescued by MCC950, indicating that ten-eleven translocation 2 mediated HF *via* an IL-1β/NLRP3 inflammasome-dependent mechanism (123). Besides, it was reported that Bay 11-7082, an inhibitor of NLRP3 inflammasome *via* suppressing the ATPase activity of NLRP3 (124), resulted in a reduction in the formation of the inflammasome in the heart and the infarct size in an I/R model when treated intraperitoneally 10 min before reperfusion (104) or 30 min before ischemia (125). Similarly, treatment with OLT1177, another NLRP3 inhibitor by blocking its ATPase activity, which was originally developed as a topical treatment for degenerative arthritis (126), also reduced the infarct area after MI/R injury (127). Recently, it has been reported that OLT1177 could preserve contractile reserve and diastolic function of the heart in a mouse MI model and may therefore be used to prevent the development of HF in patients with ischemic cardiomyopathy (128). Colchicine, administered at high doses (0.1 mg/kg per day) for 7 days in the AMI mouse model, significantly inhibited NLRP3 inflammasome expression and activation, indicated by reduced NLRP3, ASC, and caspase-1 levels in the MI area; decreased infarct size; alleviated LV remodeling; protected contractile function; and significantly delayed HF development and 7-day survival after MI (129). Acrylamide derivatives were developed, which can covalently bind to NLRP3 to inhibit its ATPase activity. A dose of 50 μmol/L of the compound given 20 min before induction of ischemia in an *ex vivo* mouse MI model reduced inflammasome activity and infarct size (130).

As a hypoglycemic agent, in addition to lowering blood sugar and reducing the incidence of cardiovascular events in diabetic patients, empagliflozin was also reported to inhibit the activation of the NLRP3 inflammasome, thus reducing cardiac inflammation and preserving cardiac function *via* a Ca^2+^-dependent mechanism (131). In consideration of the effect of the inflammasome in cardiac fibrosis, Chinese herbal medicine triptolide and active small molecule pirfenidone have been used to treat fibrosis and were shown to inhibit NLRP3 inflammasome activity, reduce macrophage infiltration and fibrosis, and improve TAC-induced cardiac diastolic and systolic functions (132, 133).

Inhibition of AIM2 with synthetic oligonucleotide A151 reduced the levels of IL-1β and IL-18 and improved plaque stability (64). Administration of probenecid, clinically used as a uricosuric drug, which blocks pannexin-1 channels, significantly reduced AIM2 inflammasome activation *in vitro*, reduced pressure overload-induced mortality, and restored indices of disease severity *in vivo*, showing that AIM2 inflammasome activation participates in chronic inflammation in HF (66).

Administration of recombinant IL-1Ra or IL-1β neutralizing antibody to *ApoE*-deficient mice significantly reduced the diet-induced formation of atheroma (36). In addition, administration of IL-18 by intraperitoneal injections into *ApoE^−/−^* mice aggravated atherosclerosis lesions (134). IL-18 neutralizing antibodies have been shown to reduce infarct size following MI/R (71), verifying a pathological role for IL-18 in MI. Daily administration of IL-18 into healthy mice for 7 days led to LV contractile dysfunction and myocardial hypertrophy (135). Correspondingly, it has been reported that treatment with recombinant IL-18 induced mouse interstitial fibrosis, diastolic dysfunction, and myocardial remodeling and increased cardiac osteopontin expression, which are components of the extracellular matrix associated with the fibrotic process during tissue remodeling (116, 136). Conversely, IL-18 neutralizing antibody protected against lipopolysaccharide-induced myocardial dysfunction in mice (137).

To sum up, pharmacological interventions with inflammasome have been shown to be beneficial in the treatment of CVDs in animal studies (**Table 2**). Further clinical studies are needed to determine whether inhibition of inflammasome can be beneficial for patients with CVDs.

**Table 2:** Pharmacological interventions of inflammasomes in animal models of CVDs.

Intervention	Animal	CVD model	Outcomes	Ref.
NLRP3 inhibitor MCC950	*Apoe^−/−^* mice	HFD for 4 weeks with semiconstrictive collar placement at the carotid arteries	Reduced the number of macrophages in the plaque and the atherosclerotic lesions	(117)
NLRP3 inhibitor arglabin	*Apoe2*.Ki mice	HFD for 13 weeks	Reduced inflammation, plasma lipids, and atherosclerotic lesions	(118)
Anti-IL-1α, anti-IL-1β, anti-IL-1α+β	*Apoe^−/−^* mice	HFD for 14 weeks and treatment with antibodies for 14 weeks	Neutralization of IL-1α or both IL-1 isoforms decreased early AS lesions and impaired outward remodeling, while IL-1β inhibition reduced the size of established atheroma	(98)
Recombinant IL-18	*Apoe^−/−^* mice	Normal diet until 20 weeks old	Increased inflammatory response and AS lesion size	(133)
NLRP3 inhibitor	CD-1 male mice	MI 30 min, R 3 or 24 h	The NLRP3 inhibitor given at reperfusion, or 1 h (but not 3 h), reduced infarct size at 24 h	(20)
NLRP3 inhibitor Bay 11-7082	C57BL/6 mice	MI 30 min, R 24 h	Decreased macrophage and neutrophil accumulation, cardiomyocyte apoptosis, and infarct size	(104)
BAY11-7082	Diabetic rats	MI 30 min, R 2 h	Reduced pyroptosis and MI/R injury in diabetic rats	(106)
BAY11-7082	Sprague–Dawley rats	MI 30 min, R 24 h or 7 days	Reduced cell apoptosis and infarct size and preserved cardiac function	(125)
NLRP3 inhibitor	Male ICR mice	MI 30 min, R 24 h, or MI 7 days	Preserved LV function, and reduced infarct size after MI or MI/R injury	(119)
NLRP3 inhibitor 16673-34-0	CD1 mice	MI 30 min, R 24 h	Limited infarct size after MI/R	(120)
NLRP3 inhibitor MCC950	Female landrace pigs	MI 75 min, R 7 days	Reduced myocardial neutrophil influx, IL-1β levels, infarct size, and preserved cardiac function	(122)
NLRP3 inhibitor OLT1177	WT mice	MI 30 min, R 24 h or 7 days	Reduced infarct size and preserved LV systolic function given within 60 min after MI/R	(127)
NLRP3 inhibitor OLT1177	WT mice	MI 7 days	Preserved cardiac contractile reserve and diastolic function	(128)
Colchicine	Male C57BL/6J mice	MI 7 days	Inhibited the expression of NLRP3 inflammasome and infiltration of neutrophils and macrophages, improved cardiac function and survival rate	(129)
IL-18-neutralizing antibody	C57BL/6 mice	MI 30 min, R 24 h	Reduced infarct size	(71)
MCC950	*Camk2d^fl/fl^* mice	Ang II infusion	Reduced macrophage accumulation and cardiac fibrosis	(111)
Triptolide	C57/BL6 mice	TAC	Inhibited NLRP3 inflammasome, attenuated TAC-induced myocardial remodeling, and improved cardiac function	(132)
Pirfenidone	Male Balb/c mice	TAC	Inhibited NLRP3 expression, attenuated myocardial fibrosis and inflammatory mediators, and increased survival rate	(133)
Recombinant IL-18	C57BL/6 mice		Induced cardiac hypertrophy and caused LV dysfunction	(135, 136)
IL-18 neutralization antibody	C57BL/6 mice	LPS-induced cardiac dysfunction	Attenuated LPS-induced myocardial dysfunction	(137)

CVDs, cardiovascular diseases; HFD, high-fat diet; MI, myocardial infarction; LV, left ventricle; MI/R, myocardial ischemia/reperfusion.

## 4 Clinical Studies on IL-1b and NLRP3 Inflammasome in Cardiovascular Diseases

A variety of studies confirmed that the innate immune responses mediated by inflammasome-dependent cytokines (like IL-1β) promote the development of CVDs such as atherosclerosis and HF (138, 139). Therefore, many clinical studies have been conducted targeting NLRP3 and IL-1β, aiming to discover new prevention and treatment methods for CVDs. The advantage of targeting NLRP3 inflammasome core components is to prevent pyroptosis, which is not affected by IL-1β and IL-18 inhibition. Importantly, the development of NLRP3 inflammasome inhibitors provides the possibility of targeting the harmful effects of NLRP3 (140).

### 4.1 Canakinumab

Canakinumab, a human IL-1β monoclonal antibody, has been approved by the Food and Drug Administration (FDA) for the treatment of systemic juvenile idiopathic arthritis and some periodic fever syndromes, including cold-heat protein-related periodicity syndromes and familial Mediterranean fever (141, 142). The efficacy of canakinumab in patients with AMI was studied in the multicenter Canakinumab Anti-Inflammatory Thrombosis Outcome Study (CANTOS) trial (143, 144) involving 10,061 patients with previous AMI (at least 30 days before enrollment) and elevated C-reactive protein (CRP) levels (>2 mg/L), which concluded that canakinumab at a dose of 150 mg significantly reduced the incidence of a recurrent cardiovascular event and hospitalizations for HF in patients with established atherosclerotic disease. CANTOS trial confirms for the first time that targeting an inflammatory mediator can significantly improve the cardiovascular outcome of AMI patients, which verifies the hypothesis of the inflammatory response of coronary heart disease and also provides a basis for the intervention targets of inflammatory response. The limitations of this study are as follows: it included patients with AMI, but it is unknown whether its conclusions can be extended to patients with stable coronary heart disease; the pathogenesis of CVDs is complex, and the intervention of one molecular target in the inflammatory pathway may not be sufficient; besides, as the upstream of the inflammatory response, inhibition of IL-1β by canakinumab-induced severe infection is an adverse effect that cannot be ignored.

### 4.2 Anakinra

The elevated circulating levels of IL-1β as well as IL-1Ra, IL-6, or CRP correlate with worsening HF symptoms and outcomes, clarifying the relevance of inflammation and HF (145, 146). Other clinical trials have also confirmed the importance of the inflammasome and IL-1 in CVDs by administration of anakinra, an IL-1 receptor antagonist. Treatment with anakinra (100 mg daily for 14 days) in patients with ST-segment elevation MI (STEMI) significantly reduced acute systemic inflammatory response and reduced incidence of adverse remodeling and HF at 3 months or long term (147, 148). The present study has several potential drawbacks. First, the number of patients enrolled is small, which is not sufficient to detect effects on clinical outcomes. Second, true baseline acquisition of the imaging studies is lacking, which was performed obtained 24 to 96 h after admission, and therefore, it is hard to account for early changes in size and contractility. A phase II clinical trial involving 182 patients with non-STEMI elevation AMI revealed that anakinra (100 mg daily for 14 days) reduced the acute inflammatory response. The clinical events at 30 days and 3 months were not affected by anakinra treatment, but surprisingly, anakinra significantly increased recurrent ischemic events after 12 months (149). The main limitation of this study is that the treatment lasted only 14 days and that little is known about the nature of late events, which led to the apparent cardiovascular adverse events 1 year later. In a study of 23 rheumatoid arthritis patients with elevated IL-1β levels, anakinra treatment with a dose of 150 mg significantly improved cardiac and vascular function (150). Consistently, it has been revealed that anakinra improved diastolic dysfunction, cardiac function, and quality of life in patients with decompensated systolic HF (151–153). Recently, it has been reported that targeting IL-6 (a downstream mediator of IL-1β) with tocilizumab in STEMI reduced microvascular obstruction, although it has no effect on the final infarct size; it was less beneficial in patients with symptom duration of <3 h (154), suggesting the importance of the timing of the disease course in which the inflammasome is targeted.

### 4.3 Colchicine

Colchicine was originally used to treat Mediterranean fever and was later approved by the FDA for the treatment of acute gouty arthritis because it was found to inhibit the assembly and activation of NLRP3 inflammasome with the stimulation of monosodium urate crystals (155). Colchicine inhibits the activity of NLRP3 inflammasome *via* two aspects: inhibition of P2X7 receptor activation and the polymerization of ASC by interfering with the interaction between the pyrin domains (155). Colchicine was found to inhibit mitochondrial transport and subsequent co-localization of ASC to NLRP3, ultimately suppressing the activation of NLRP3 inflammasome (156). Colchicine plays an anti-inflammatory role mainly through inhibiting NLRP3 inflammasome.

In a clinical trial (157), patients with stable coronary artery diseases were randomly treated with a low dose of colchicine (0.5 mg daily) or placebo, for a median time of 2.3 years. Compared with placebo, colchicine significantly reduced the incidence of adverse cardiovascular endpoints, assessed by reduction of the incidence of acute coronary syndrome. But this trial mainly focused on the effects of colchicine on non-fatal cardiovascular events. Despite the trend toward improved mortality, larger studies are needed to confirm whether colchicine may reduce the risk of fatal cardiac events. It would make sense to investigate the value of colchicine in patients recently hospitalized for the acute coronary syndrome, as they remain at high risk of recurrent events due to plaque disruption (158). Consistent with the important role of NLRP3 inflammasome in ischemia–reperfusion injury, in phase II clinical trial, colchicine (initial dose 1.5 mg, 0.5 mg once or twice daily according to body weight) significantly reduced the infarct size of patients with STEMI and inflammatory biomarkers undergoing primary percutaneous coronary intervention (159). However, the limitation of this study is that 26% of the 77 patients in the colchicine group discontinued treatment due to poor tolerance, which is common when colchicine doses exceed 1.0 mg/day. These clinical studies clarify the contributive role of NLRP3 inflammasome in AMI and HF, which may be a priming target. Conversely, the latest clinical trial enrolled in patients with STEMI referred for primary percutaneous coronary intervention suggested that high-dose colchicine at the time of reperfusion and for 5 days (2-mg loading dose followed by 0.5 mg twice a day) did not reduce the infarct size and LV remodeling (160). The inconsistent result may be because in the former study (159), infarct size reduction was assessed by the release of myocardial biomarker, and only a subgroup of patients was assessed on cardiac MRI. Another explanation may be that in the former study, patients’ thrombosis in MI flow was not reported, suggesting potential confounding factors. More reliable evaluation criteria and primary endpoint events in larger studies are needed in the future.

In summary, clinical data suggest that the IL-1 cytokine family contributes to cardiac dysfunction and that blocking IL-1 with anakinra or canakinumab improves cardiac function and prevents hospitalization in patients with HF. Inhibition of NLRP3 inflammasome protects against myocardial injury in AMI and adverse cardiac remodeling in HF. So far, these data are the strongest evidence that the NLRP3 inflammasome and IL-1β play an important role in CVDs (144, 145) (**Table 3**).

**Table 3:** Clinical trials of NLRP3 inflammasome and IL-1 or IL-6 blockers in CVDs.

Clinical drugs	Indication (n)	Study design	Outcomes	Refs
Canakinumab (2017)	Patients with previous AMI at least 30 days and undergoing revascularization, whose high-sensitivity CRP > 2 mg/L (10061)	Randomized, canakinumab 50, 150, or 300 mg, or placebo subcutaneously given every 3 months for a median follow-up of 3.7 years	Reduced high-sensitivity CRP levels without reducing the LDL level; Reduced the incidence of primary endpoint of cardiac death, non-fatal AMI at dose of 150 mg	(144)
Canakinumab (2019)	Patients with prior myocardial infarction and high CRP > 2 mg/L (10061)	Randomized, canakinumab 50,150,300 mg, or placebo subcutaneously given every 3 months for a median follow-up of 3.7 years	A dose-dependent reduction in the composite of hospitalization for heart failure and heart failure-related mortality	(145)
Anakinra (2013)	Patient with stable STEMI (30)	Randomized, anakinra or placebo subcutaneously given 100 mg/day for 14 days for a follow-up of 10-14 weeks	Blunted the acute inflammatory response; led to a lower incidence of heart failure	(147)
Anakinra (2015)	Patient with stable STEMI (40)	Randomized, anakinra or placebo subcutaneously given 100 mg/day for 14 days for a follow-up of 28 months	A neutral effect on recurrent ischemic events; may prevent new-onset heart failure	(148)
Anakinra (2015)	Patient with non-ST elevation ACS presenting <48 h from onset of chest pain (182)	Randomized, IL-1Ra (anakinra) or placebo subcutaneously given 100 mg/day for 14 days for a follow-up of 12 months	Reduced high-sensitive CRP but rose again at 30 days; major adverse cardiovascular events had no difference at 30 days or 3 months but increased at 1 year	(149)
Anakinra (2008)	Patients with rheumatoid arthritis (23:19)	Non-randomized anakinra 150 mg/day or prednisolone subcutaneously given for 30 days	Improved biomarkers and vascular and LV function after 30 days of treatment	(150)
Anakinra (2017)	Patients with reduced LV ejection fraction (<50%) and elevated CRP levels (>2 mg/L), within 14 days of hospital discharge (60)	Randomized, anakinra or placebo subcutaneously given 100 mg/day for 2 and 12 weeks with a follow-up of 24 weeks	Improved peak oxygen consumption and reduced the incidence of death or rehospitalization	(151)
Anakinra (2016)	Patients with acute decompensated heart failure, reduced LV ejection fraction (<40%), and elevated CRP levels (>5 mg/L) (30)	Randomized, anakinra or placebo 100 mg subcutaneously given twice daily for 3 days followed by once daily for 11 days	Reduced levels of CRP, IL-6, and systemic inflammatory response	(152)
Tocilizumab (2021)	Patients with STEMI within 6 h of symptom (199)	Randomized, a single intravenous infusion of 280 mg of tocilizumab or placebo with a follow-up of 6 months	Reduced microvascular obstruction, although no effect on the final infarct size	(154)
Colchicine (2011)	Patients with clinically stable coronary disease for at least 6 months (532)	Randomized, colchicine 0.5 mg or placebo given daily for a median follow-up of 3 years	Colchicine 0.5 mg/day in addition to statins and other secondary prevention therapies reduced the risk of cardiovascular events	(158)
Colchicine (2015)	Patients with STEMI ≤12 h from pain onset (151)	Randomized, colchicine given a loading dose of 2 mg, and continued with 0.5 mg twice daily, or placebo, for 5 days	Reduced the relative infarct size and the incidence of acute coronary syndrome	(159)
Colchicine (2021)	Patients with STEMI referred for primary percutaneous coronary intervention (192)	Randomized, colchicine or placebo given a 2-mg loading dose followed by 0.5 mg twice daily, for 5 days with a follow-up of 3 months	No significant difference in infarct size and LV remodeling between the colchicine and placebo groups	(160)
OLT1177 (2021)	Patients with heart failure and reduced ejection fraction (30)	Randomized, OLT1177 at dose of 500, 1,000, and 2,000 mg for up to 14 days, each including 10 patients	OLT1177 was safe and well tolerated, and LV ejection fraction improved significantly in the 2,000-mg group	(161)

CVDs, cardiovascular diseases; AMI, acute myocardial infarctions; CRP, C-reactive proteins; LDL, low-density lipoproteins; STEMI, ST-segment elevation myocardial infarction.

### 4.4 OLT1177

Recently, the effect and safety of an oral inhibitor of the NLRP3 inflammasome, OLT1177, have been studied in patients with HF and reduced ejection fraction. Subjects were randomized to treatment with OLT1177 for up to 14 days at ascending dose cohorts (500, 1,000, and 2,000 mg), each including 10 patients, and underwent clinical assessment at baseline, day 14, and day 28. Results showed that treatment with OLT1177 for 14 days was safe and well tolerated, and LV ejection fraction improved significantly in the 2,000-mg group (161), suggesting that NLRP3 inhibition can be an important therapeutic target for HF and provide preliminary safety evidence.

CANTOS study is the first successful large-scale anti-inflammatory therapy of CVDs in the clinic and enriches the theoretical system of atherosclerotic CVDs, which is a supplement and improvement to the traditional cholesterol theory. Traditional medicines such as statins and PCSK9 inhibitors reduce cardiovascular risk and play a protective role mainly by lowering blood lipid levels. However, many patients who still have high levels of hypersensitive CRP after statin therapy, indicating a high risk of residual inflammation, often have a poor clinical prognosis. CANTOS study suggests that for these patients, further anti-inflammatory therapy in addition to statin therapy may be considered to maximize cardiovascular benefit.

Most acute coronary events are associated with the rupture of unstable atherosclerotic plaques, and inflammation induced by inflammasome is an important cause of plaque instability and rupture (36, 37). Clinical studies targeting the NLRP3 inflammasome have achieved preliminary clinical benefits in cardiovascular patients, also broadened the targets of inflammatory intervention, and enriched the inflammatory hypothesis. Interestingly, necessary anti-inflammatory therapy with anakinra in rheumatoid arthritis patients also reduced cardiovascular risk and improved cardiac function, further confirming the cardiovascular protective effect of anti-inflammatory therapy.

However, current clinical studies still have some limitations. Clinical trials targeting anakinra and colchicine mainly focused on AMI but few on other types of CVDs; in addition, the scale of most clinical studies is small, and evidence of therapeutic effect in a larger population of CVDs patients is lacking. Whether inhibition of inflammasomes and downstream effectors (such as NLRP3 and IL-1β) can be an effective clinical therapy for the treatment of CVDs needs to be confirmed in larger clinical trials with more inhibitors.

## 5 Conclusion

In conclusion, a variety of danger signals and pathogenic factors can trigger the priming and activation of the inflammasomes. The activated inflammasomes promote the production and release of the inflammatory cytokines and the systemic inflammatory response, thus playing a key role in the pathological process of CVDs, such as atherosclerosis, myocardial ischemic and non-ischemic injury, and HF. The inflammasome is a promising therapeutic target for CVDs. Therefore, the process of activation and assembly and the precise regulatory mechanism in cell pyroptosis and the progression of CVDs of the inflammasomes are worthwhile to be further studied in the future.

A lot of animal experiments and clinical trials confirm that inflammasomes and the cytokine family, especially NLRP3 inflammasome and IL-1, are central to the pathological response to injury. Therefore, NLRP3 and IL-1 are considered promising targets for CVDs. However, no selective NLRP3 inhibitors are clinically available at present, and the clinical possibilities of IL-1 blockers are being explored. Specific oral NLRP3 inflammasome inhibitors are in clinical development. It will be of great value to the clinic to develop novel inhibitors of inflammasome and proinflammatory cytokines. In the future, there may be inhibitors that target the IL-1 subtype, possibly as well as oral NLRP3 inflammasome inhibitors for a wide range of CVDs (162).

It is worth noting that the optimal timing for administering drugs targeting the inflammasome remains unclear, which depends on the specific drug, type, and severity of CVDs. Future research should also aim to map the oscillations of drug targets such as IL-1β throughout the day so that drugs can be administered at the most effective times (163). Drug target, dose, dosage form, time point, and duration of administration will be the focus of future research to maximize the benefit to the patient.

## Author Contributions

YL and LZ are responsible for writing and revising this review. KL participated in the revision of the review. All authors listed have made a substantial, direct, and intellectual contribution to the work and approved it for publication.

## Funding

This work was supported by the National Natural Science Foundation of China (82100441).

## Conflict of Interest

The authors declare that the research was conducted in the absence of any commercial or financial relationships that could be construed as a potential conflict of interest.

## Publisher’s Note

All claims expressed in this article are solely those of the authors and do not necessarily represent those of their affiliated organizations, or those of the publisher, the editors and the reviewers. Any product that may be evaluated in this article, or claim that may be made by its manufacturer, is not guaranteed or endorsed by the publisher.

## Abbreviations

CVDs, cardiovascular diseases; HF, heart failure; MI, myocardial infarction; AS, atherosclerosis; MI/R, myocardial ischemia/reperfusion; DAMPs, damage-associated molecular patterns; IL-1β, interleukin-1beta; NLRP, NOD, LRR, and PYD domain-containing protein; NLRC, NOD, LRR, and CARD domain-containing protein; AIM2, absent in melanoma 2; ASC, apoptosis-associated speck-like protein containing CARD; PYD, pyrin domain; CARD, caspase recruitment domain; P2X7, purinoreceptor 7; AMI, acute myocardial infarction; GSDMD, gasdermin D; ROS, reactive oxygen species; ChCs, cholesterol crystals; ox-LDL, oxidized low-density lipoprotein; oxPLs, oxidized phospholipids; ABCA1/G1, ATP-binding cassette A1 and G1; ICAM-1, intracellular adhesion molecule-1; IL-1Ra, IL-1 receptor antagonist; LV, left ventricle; TAC, transverse aortic constriction; CANTOS, Canakinumab Anti-Inflammatory Thrombosis Outcome Study; CRP, C-reactive protein; STEMI, ST-segment elevation myocardial infarction.
